# *Ophioderma peruana*, a new species of brittlestar (Echinodermata, Ophiuroidea, Ophiodermatidae) from the Peruvian coast

**DOI:** 10.3897/zookeys.357.6176

**Published:** 2013-12-02

**Authors:** Tania Pineda-Enríquez, Francisco A. Solís-Marín, Yuri Hooker, Alfredo Laguarda-Figueras

**Affiliations:** 1 Colección Nacional de Equinodermos “M. Elena Caso M.”, Laboratorio de Sistemática y Ecología de Equinodermos, Instituto de Ciencias del Mar y Limnología, Universidad Nacional Autónoma de México, Ciudad Universitaria s/n Deleg. Coyoacán CP 04510 México; 2 Laboratorio de Biología Marina, Facultad de Ciencias y Filosofía, Universidad Peruana Caytano Heredia, Av. Honorio Delgado 430, Urb. Ingeniería, S.M.P. Lima, Perú

**Keywords:** Taxonomy, Ophiodermatidae, *Ophioderma peruana*, new species

## Abstract

*Ophioderma peruana* **sp. n.** is a new species of Ophiodermatidae, extending the distribution of the genus *Ophioderma* to Lobos de Afuera Island, Peru, easily distinguishable from its congeners by its peculiarly fragmented dorsal arm plates. Dense granules, rounded or polygonal cover the disc, the radial shields may be naked or completely covered by granules. A good character for recognizing this species in the field is the dorsal side of the disc which is brown with disc granules lighter cream and brown, the arms are mottled with whitish spots and the ventral part of the disc on the interradial part is brown and the radial part bright yellow.

## Introduction

Species of *Ophioderma* have a distinctive shape and color and are distributed across the Mediterranean Sea, Atlantic Ocean and off the American Pacific coast. The genus *Ophioderma* has had an interesting taxonomic history since 1840 when Müller & Troschel, described the genus, being the type species *Ophioderma longicauda* (Bruzelius, 1805) recognized by H.L. Clark in 1915. In the past, the classification of *Ophioderma* has been unstable, classified as the genus *Ophiura*, in the 19^th^ century (e.g. Lyman 1860, 1882). The first taxonomist to arrange the numerous species under the name *Ophioderma* was H.L. Clark (1915), A.M. Clark (1976) and Melville (1980), formally separating the previously controversial genus *Ophiura* from *Ophioderma*. However, Ziesenhenne (1955) made the latest revision of the genus *Ophioderma* when 21 species were known (Stöhr et al. 2009). *Ophioderma* is now well-established and comprises a large, widespread genus of brittlestars. Up to until now, *Ophioderma* comprises 27 species, 21 of which are distributed in the Atlantic Ocean and six in the Pacific Ocean. *Ophioderma* can be found in coral reefs, seagrass, coral rubble and under rocks and typically found together with other shallow-water genera, such as *Ophiocoma*, *Ophiothrix*, *Ophiolepis* and *Ophiactis*. Bathymetric distribution of the genus extends from shallow water to 50 m and is restricted to tropical and temperate seas. The characters used to separate the species are the shape of the disc granules, the disc size, arm length, shape and degree of fragmentation of dorsal arm plates, number of arm spines and color (Stöhr et al. 2009). However, the genus remains poorly studied; for example, it has been recently discovered that the species *Ophioderma longicauda* shows cryptic speciation and represents a species complex (Stöhr et al. 2009, Boissin et al. 2011).

As part of a program since 1999 to sample the coast of Peru in order to discover new or previously unreported echinoderm species, different localities have been sampled along the coastline and adjacent islands (from littoral to 30 m depth). At Lobos de Afuera Islands, Lambayeque, Peru (06°55'5"S, 80°42'5"W) a total of 39 echinoderm species have been reported, including six ophiuroids (*Ophiactis mirabilis*, *Ophiothrix spiculata*, *Ophiocoma aethiops*, *Ophioderma panamensis* and *Ophionereis annulata*), one of which is described in this paper as a new species of *Ophioderma* (Hooker et al. 2005).

Peruvian echinoderms are represented by 215 species: Crinoidea (1 species), Asteroidea (64 species), Ophiuroidea (42 species), Echinoidea (35 species) and Holothuroidea (73 species) (Hooker et al. 2013). Only two species belonging to the genus *Ophioderma* have been reported for Peruvian waters: *Ophioderma panamensis* Lütken, 1859 and *Ophioderma teres* (Lyman, 1860). Even though the new species, is easily distinguishable from its congeners, by the number of fragmented dorsal arm plates.

## Materials and methods

**Figure 1.**
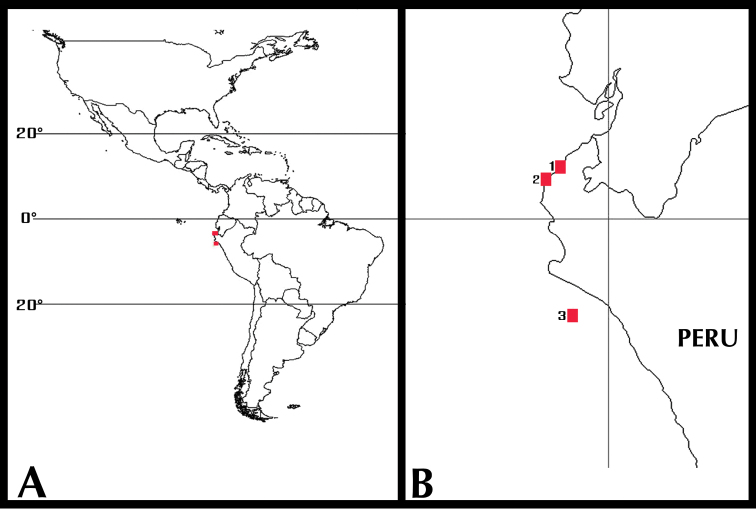
Collecting sites of *Ophioderma peruana* sp. n. **A** complete map of America **B1** Hooker Reef, Punta Sal **2** Quebrada Verde, El Ñuro **3** Lobos de Afuera Island, Peru.

Samples were taken by SCUBA in the intertidal zone at Lobos de Afuera Island, Lambayeque, Peru (Fig. 1) due to its complexity and it’s southernmost limit for many echinoderm species (Hooker et al. 2005). After collection, specimens were placed inside plastic bags with seawater for transportation. The animals were then relaxed in a solution of 4% magnesium chloride and seawater. After labeling, fixation of the specimens was done with 70% ethyl alcohol. Some specimens were dried and photographed in the laboratory under a SZ-ST Olympus dissecting microscope. Holotype and paratypes have been preserved in alcohol and dried, respectively. The specimens are deposited at the Colección Nacional de Equinodermos “M. Elena Caso M.” of the Instituto de Ciencias del Mar y Limnología, Universidad Nacional Autónoma de Mexico (UNAM–ICML) and in the Laboratorio de Biología Marina, Facultad de Ciencias y Filosofía, Universidad Peruana Caytano Heredia, Lima, Peru (CZA). Abbreviations used in this paper are: DD: disc diameter, AL: arm length; AW: arm width.

Only 13 specimens of the new species of *Ophioderma* were collected on different localities along the Pacific coast, but they are sufficiently distinctive and unique to establish a new species.

## Taxonomy

### Family Ophiodermatidae Ljungman, 1867

#### Genus Ophioderma

Müller & Troschel, 1840

http://species-id.net/wiki/Ophioderma

##### Type species.

*Ophioderma longicauda* (Bruzelius, 1805).

##### Diagnosis.

(modified from Müller and Troschel 1840) The dorsal and ventral surfaces of the disc are covered by granules. Sometimes these granules cover the radial and adoral shields. Oral papillae are broader than long, rectangular or conical in shape. There are three to five teeth. The oral shields are oval, pentagonal or triangular in shape. Each interradial space has four genital slits; the first two are on the distal side of the oral shield and the second are parallel to the arms and near the disc edge. The arms are cylindrical proximally and conical distally. The dorsal arm plates are broader than long and can be fragmented. The lateral arm plates are semi-lunar in outline and have six to thirteen arm spines that are large, rectangular or conical in shape. There are two tentacle scales per segment.

##### Remarks.

There are six known species of the genus reported for the eastern and southern Pacific in addition to the new species described here.

#### Ophioderma peruana

Pineda-Enríquez, Solís-Marín, Hooker & Laguarda-Figueras sp. n.

http://zoobank.org/10BFEA05-4299-4C52-9B0C-89CCAC178E9C

http://species-id.net/wiki/Ophioderma_peruana

##### Type specimen.

Holotype, CZA-363, Lobos de Afuera Island, Peru, 6°56'16.8"S, 80°43'22.7"W, intertidal, under rocks, October 9th, 2007.

##### Type locality.

Peru: Lobos de Afuera Island, 6°56'16.8"S, 80°43'22.7"W, intertidal, under rocks, October 9th, 2007.

##### Other type material.

**Figure 2.**
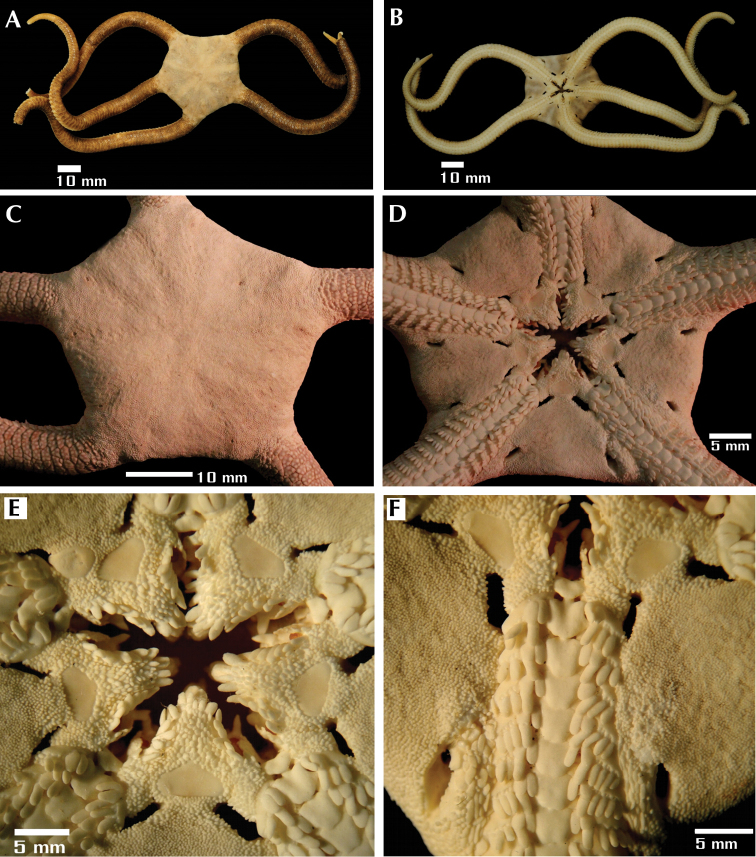
*Ophioderma peruana* sp. n., holotype (CZA-363). **A** aboral view **B** oral view **C** aboral disc and basal portion of the arms **D** oral disc and basal portion of the arms **E** jaws **F** oral portion of the disc and pair of genital slits.

Paratype, CZA-364, Lobos de Afuera Island, Peru, 6°56'16.8"S, 80°43'22.7"W, intertidal, October 9th, 2007; paratype, CZA-365, Lobos de Afuera Island, Peru, 6°56'16.8"S, 80°43'22.7"W, intertidal, October 9th, 2007; paratype, UNAM-ICML 3.234.0, Lobos de Afuera Island, Peru, 6°56'16.8"S, 80°43'22.7"W, intertidal, under rocks, October 9th, 2007 (Fig. 2).

##### Diagnosis.

Disc pentagonal, elevated and covered with dense granules that are somewhat rounded or polygonal, but more or less dispersed. The radial shields can be completely covered by the granules or scarcely covered. The dorsal arm plates are fragmented; in addition there are some smaller and tiny fragments that resembles granules of the dorsal disc, only visible on the proximal plates (not evident in all the arm segments). Nine or ten flattened and elongated oral papillae. Granules also cover the adoral shields. Ten arm spines, the ventral is the largest, reaching the next tentacle scale.

##### Description of holotype.

CZA-363: disc diameter 36.3 mm, arm length 120.6 mm, arm width 7.6 mm (Fig. 2).

**Disc.** Disc pentagonal, broad and flat, covered by granules; the dorsal granules are closely packed and have the same size on the middle and periphery of the disc, these granules are rounded and polygonal. The radial shields are almost fully covered by granules with only a small section exposed; the size is 3.81 mm and fit 9.5 times the disc diameter; the disc scales are small and imbricated, oval shape with polygonal borders, the interradial scales are smaller than the radial ones. Jaws with seven to nine oral papillae; the two distal ones are stout and longer than broad. The oral papillae have rounded edges and are almost of the same size and shape. The oral shields are broader than long, triangular in shape with convex proximal sides and are surrounded by granules that are slightly larger than those on the interradial disc surface. The adoral shields are rectangular and covered by larger and taller granules than those on the dorsal disc, which are contiguous. Four genital slits on each interradii; the two proximal ones are touching the oral shield and are located between the distal part of the oral shields and the first lateral arm plate; the two distal genital slits are placed between the fifth and and the sixth arm segment and close to the periphery of the disc.

**Figure 3.**
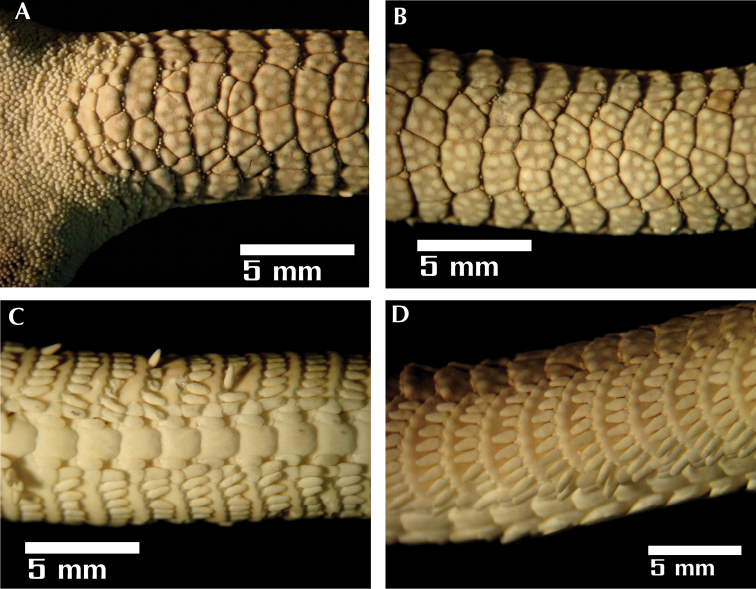
*Ophioderma peruana* sp. n., holotype (CZA-363). **A** basal portion of the arms with fragmented dorsal arm plates **B** dorsal arm plates fragmented in several pieces **C** ventral arm plates and tentacle scales **D** lateral view of the arm spines.

**Arms.** The basal portion of the arm is 7.6 mm broad and the arm length is 120.6 mm. The dorsal arm plates occupy less than 1/4 of the arm, are 4.6 times wider than long and rectangular, fragmented in six pieces that differ in shape; there are some granules on the proximal portion and sparcely distributed on the distal portion. The lateral arm plates have a half-circle shape, and occupy a sub-ventral position; with ten arm spines conical, large and slightly flattened with a rounded tip, half segment length decreasing slightly in size dorsally. The ventral-most arm spine is the longest and widest, almost the size of the segment. The ventral arm plates are contiguous, broader than long, the proximal plates are elongated in comparison to the distal plates. Two tentacle scales on each side of the ventral plate; the adradial tentacle scale is oval in shape, twice as long as wide and the abradial tentacle scale triangular in shape, with the straight side touching the ventral arm plate (Fig. 3).

**Color.** Specimen preserved in alcohol. The dorsal side of the disc is light brown and the arms are darker brown, the dorsal arm plates of each segment are ornamented with a double row of tiny, whitish, rounded spots; the spines are brown except the two ventral ones that are cream color, like the ventral side of the arms; the jaws are white; the ventral side of the disc in the proximal part is white and the distal part is slightly darker; the oral shields are mottled. Dry specimens, have the dorsal side of the disc pale brown, the arms are brown with black and white spots; the tentacle feet are yellowish. Live specimens in the field could be identified by this color pattern: the dorsal side of the disc is brown with the disc granules lighter cream and brown; the arms are mottled with whitish spots; the ventral disc interradii are brown and arms under the disc are bright yellow.

**Figure 4.**
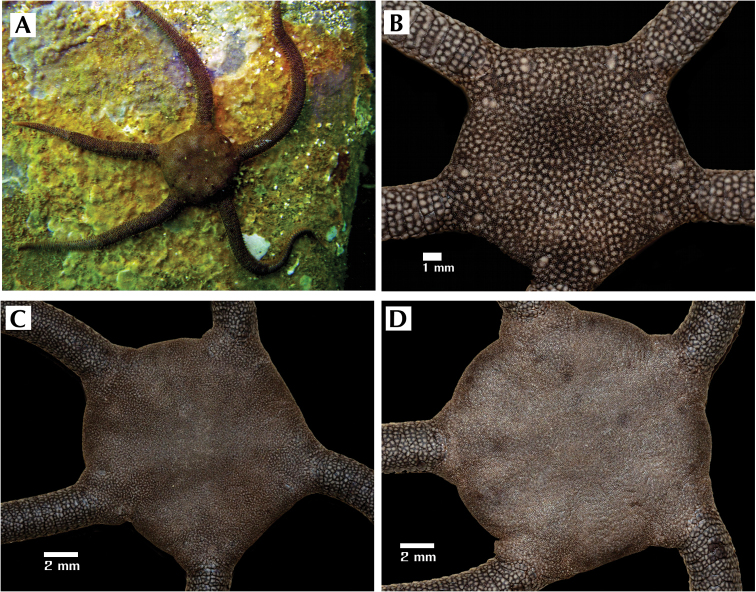
*Ophioderma peruana* sp. n.non-type preserved material (alcohol) showing different colors variations. **A** specimen *in situ* **B** CZA-394 (14 mm DD) radial shields naked **C** CZA-392 (28 mm DD) radial shields naked **D** CZA-390 (35 mm DD) radial shields covered by disc granules.

**Paratype variations.** Onthe smallest specimen (14 mm DD; 35 mm AL; 4 mm AW) the radial shields are completely naked with white spots (same color pattern as the dorsal arm plates), oval and surrounded by the disc granules by the disc granules, scarcely covered (in specimens with 40–42 mm DD) or completely naked (in specimens with14–35 mm DD). On certain segments of the arm, the dorsal arm plates are not as fragmented, with only two or three pieces. The presence of granules along the arm is not evident as in the holotype. In some specimens (22–31 mm DD) the radial shields are also completely naked. The oral shields are twice as wide as long, proximally elongated but the shape may vary in specimens. In two specimens (30 mm; 42 mm DD) the radial shields are naked and/or covered by granules. The radial shields are completely covered by granules and dorsal arm plates are fragmented in only a single specimen (35 mm DD) (Fig. 4). Therefore, as the animal grows, the radial shields become more covered in granules and the dorsal arm plates are fragment further.

##### Distribution.

Only known from the coast of Peru. Lobos de Afuera Island, Lambayeque, Peru; intertidal (type locality); Quebrada Verde, El Ñuro, Peru, 9 m; 4°13'39.3"S, 81°12'30.0"W and Hooker Reef, Punta Sal, Peru; 14 m; 3°57'14.20"S, 80°57'48.50"W (Fig. 1).

##### Etymology.

Named after the type locality.

##### Remarks.

The new species is distinguishable by its thick and rounded granules on the disc, the number of fragments of the dorsal arm plates, which can be more than six with other smaller fragments. The distal border of the dorsal arm plates, from the base to the middle part of the arm, supports some granules similar to those on the dorsal part of the disc.

In Peruvian waters, *Ophioderma panamensis* and *Ophioderma teres* are found on the same localities, in addition tothe new species; it differs from other Peruvian species in shape and size of the tentacle scales and in the shape of the arm spines. It differs from *Ophioderma teres* by the smaller size and density of the granules on the disc. These granules are similar to those present on *Ophioderma sodipallaresi*, the main difference is that on the latter species they are somewhat more scattered than in *Ophioderma teres*, while in *Ophioderma peruana* sp. n. the dorsal granules are closely packed and have the same size on the middle and periphery of the disc, being rounded and polygonal. *Ophioderma sodipallaresi* differs in havingonly two to three fragments, whereas *Ophioderma teres* have a similar number to *Ophioderma peruana* sp. n. The shape and size of the tentacle scales in *Ophioderma teres* are similar to *Ophioderma peruana* sp. n., oval and elongated, whereas in *Ophioderma sodipallaresi* the abradial tentacle scale is longer than wide and the adradial scale is smaller, almost triangular or oval. The ventralmost arm spines are largest in all three species, and the others increase in size from dorsal to ventral. In *Ophioderma peruana* sp. n. the arm spines are thick and conical, similar to *Ophioderma sodipallaresi*, which are pointed, thick and short, but differing in size. Meanwhile, in *Ophioderma teres* the arm spines are almost flat with pointed tips. In comparison with the other West Pacific ophiodermatids species, *Ophioderma panamensis* and *Ophioderma vansyoci* differs from *Ophioderma peruana* sp. n. by presenting the radial shields naked, just bordered by the granulation of the disc; in contrast with *Ophioderma variegata* and *Ophioderma pentacantha* that has the radial shields covered by the disc granules, while in *Ophioderma peruana* sp. n. the radial shields could be naked or covered by the granules. *Ophioderma vansyoci* presents the dorsal arm plates fragmented in three pieces. The number of arm spines are variable, *Ophioderma pentacantha* has five, *Ophioderma vansyoci* has seven, *Ophioderma panamensis* and *Ophioderma variegata* has eight, while *Ophioderma peruana* sp. n. presents ten, *Ophioderma sodipallaresi* seven arm spines and *Ophioderma teres* nine arm spines. *Ophioderma variegata* and *Ophioderma pentacantha* presents the adoral shields slightly naked, in comparison with *Ophioderma panamensis*, *Ophioderma vansyoci*, *Ophioderma sodipallaresi*, *Ophioderma teres* and *Ophioderma peruana* sp. n. that presents the adoral shields covered by the disc granules. Among its congeners in the Caribbean Sea, *Ophioderma peruana* sp. n. is more similar to *Ophioderma squamosissima* and *Ophioderma guttata* sharing fragmented dorsal arm plates (more than six pieces) but differs from the later ones in the absence of the smaller scales on the dorsal arm plates, by having different shape of disc granules (rounded and polygonal in *Ophioderma peruana* sp. n., flattened, elongated and polygonal shape in *Ophioderma squamosissima* and flattened, shorter and polygonal in *Ophioderma guttata*), in addition to its geographic distribution. The rest of the *Ophioderma* species distributed in the Caribbean Sea either lacks fragmented arm plates (*Ophioderma appressa*, *Ophioderma brevicauda*, *Ophioderma brevispina*, *Ophioderma phoenium* and *Ophioderma rubicunda*) or some segments of the dorsal arm plates could be fragmented (*Ophioderma cinerea*).

### Key to the Pacific Ocean species of *Ophioderma*

**Table:** 

1	Radial shields either naked or covered by granules, disc and dorsal side of the arms mottled	2
–	Radial shields always naked, disc and dorsal side of the arm not mottled	6
2	Dorsal arm plates fragmented	3
–	Dorsal arm plates not fragmented	5
3	Nine to ten arm spines, the ventral-most thicker than long, somewhat flat; oral shields triangular-shaped; adoral shields covered by granules; tentacle scales subequal, oval-shaped	*Ophioderma teres* Lyman, 1860
–	The pair of tentacle scales are of different size	4
4	Nine arm spines, the ventralmost is the largest and pointed; oral shields slightly pentagonal in shape, the adoral shields covered by granules; the dorsal arm plates are fragmented into two or three pieces; disc granules are somewhat more scattered oval-shape; radial shields covered by granules; the abradial tentacle scale is longer than wide	*Ophioderma sodipallaresi* Caso, 1986
–	Ten arm spines, the ventralmost longest; oral shields triangular; adoral shields covered by granules, dorsal arm plates completely fragmented (more than six pieces), disc granules oval-shaped, densely placed; radial shields naked or covered by granules; the abradial tentacle scale is two times longer than wide	*Ophioderma peruana* sp. n.
5	Arms three times disc diameter; oral shields oval-shaped, longer than wide; adoral shield naked; eight arm spines	*Ophioderma variegata* Lütken, 1856
–	Arms five times disc diameter; radial shields covered in granules; oral shields pentagonal; adoral shields naked; five short arm spines half the length of the arm segment, the ventral-most is wider than long	*Ophioderma pentacantha* H. L. Clark, 1917
6	Dorsal arm plates not fragmented; arms three times disc diameter; radial shields completely naked; oral shields wider than long; adoral shields covered by granules; eight arm spines	*Ophioderma panamensis* Lütken, 1859
–	Dorsal arm plates fragmented into three pieces; disc granules flattened; radial shields convex and naked; oral shields pentagonal; adoral shields covered by granules; seven arm spines	*Ophioderma vansyoci* Hendler, 1996

## Discussion

The new species clearly belongs to the genus *Ophioderma* Müller & Troschel, 1840. Its large size makes it a conspicuous component of the eastern Pacific shallow-water echinoderm fauna. *Ophioderma peruana* sp. n. has been collected at the same sites as one of its congeners, *Ophioderma panamensis*.

Presently 28 valid species and two highly doubtful species (*Ophioderma propinqua* Koehler, 1895 and *Ophioderma tongana* Lütken, 1872) should now be recognized as part of the genus *Ophioderma*. The genus is well wide-spread, but most speciose in the western Atlantic Ocean with 18 species, from New York, USA, to the coast of Brazil: *Ophioderma anitae* Hotchkiss, 1982, *Ophioderma appressa* (Say, 1825), *Ophioderma besnardi* Tommasi, 1970, *Ophioderma brevicauda* Lütken, 1856, *Ophioderma brevispina* (Say, 1825), *Ophioderma cinerea* Müller & Troschel, 1842, *Ophioderma devaneyi* Hendler & Miller, 1984, *Ophioderma divae* Tommasi, 1971, *Ophioderma elaps* Lütken, 1856, *Ophioderma ensifera* Hendler & Miller, 1984, *Ophioderma guttata* Lütken, 1859, *Ophioderma holmesii* (Lyman, 1860), *Ophioderma januarii* Lütken, 1856, *Ophioderma pallida* (Verrill, 1899), *Ophioderma phoenium* H.L. Clark, 1918, *Ophioderma rubicunda* Lütken, 1856, *Ophioderma squamosissima* Lütken, 1856.

In the Indian Ocean *Ophioderma wahlbergii* Müller & Troschel, 1842, has been recorded, while *Ophioderma longicauda* (Bruzelius, 1805) occurs in the Mediterranean Ocean and some eastern Atlantic localities.

In the Pacific Ocean there are seven species, from California, USA to the coast of Chile: *Ophioderma panamensis* Lütken, 1859, *Ophioderma pentacantha* H.L. Clark, 1917, *Ophioderma sodipallaresi* Caso, 1986, *Ophioderma teres* (Lyman, 1860), *Ophioderma vansyoci* Hendler, 1996, *Ophioderma variegata* Lütken, 1856, and *Ophioderma peruana* sp. n.

Only one species of fossil *Ophioderma* has been described, *Ophioderma bonaudoae* Martinez & Del Rio, 2008, from the late Miocene of Argentina.

*Ophioderma peruana* sp. n. is the third record of a species of *Ophioderma* for the Tropical Peruvian Eastern Pacific. It is the second *Ophioderma* species reported from Lobos de Afuera Island, Quebrada Verde, El Ñuro and Hooker Reefs. The Peruvian sea is considered one of the most productive in the world because of an intense upwelling system off most of its coastline (Hooker et al. 2013). It is important to continue the research in this area, because besides being the southern limit of distribution of Panamic fauna, the remote Lobos de Afuera Islands could be an area of endemism, due to less frequent dispersal from the mainland (Hooker et al. 2005).

*Ophioderma propinqua* Koehler, 1895, and *Ophioderma tongana* Lütken, 1872, have been described from the Indo-west Pacific. With the exception of the mistaken identification of *Ophioderma tongana* from Simon’s Bay, South Africa (see Mortensen 1933; A.M. Clark and Rowe 1971, A.M. Clark and Courtman-Stock 1976), these two species have not been reported in over 100 years since they were first described. Their validity is therefore doubtful and their identification within the genus *Ophioderma* must be considered suspect.

## Supplementary Material

XML Treatment for Ophioderma

XML Treatment for Ophioderma peruana
